# The effect of the simulated intrauterine sound on behavioral and physiological indices of pain during capillary blood sampling for screening preterm infants: a randomized clinical trial study

**DOI:** 10.1186/s12887-024-04604-6

**Published:** 2024-02-13

**Authors:** Shamimeh Yarahmadi, Moluk Pouralizadeh, Zahra Atrkarroushan, Parichehr Shahroudi

**Affiliations:** 1 Department of Nursing, School of Nursing and Midwifery, Guilan University of Medical Sciences, Rasht, Iran; 2 Department of Biostatistics, Medical School, Guilan University of Medical Sciences, Rasht, Iran; 3 Beheshti School of Nursing and Midwifery, Guilan university of Medical Sciences, Hamidyan Ave, Rasht, Iran

**Keywords:** Preterm infant, Blood specimen, Pain, Heel, Voice

## Abstract

**Introduction:**

Due to medical procedures, preterm infants are at high risk for side effects of pain. In this regard, heel lancing for capillary blood sampling is a common painful procedure. The present study was conducted to assess the effectiveness of a simulated intrauterine sound on behavioral and physiological indices of pain due to heel-prick blood sampling in preterm infants.

**Methods:**

A double‑blind randomized clinical trial (RCT) was conducted. The data were collected from September 23 to December 22, 2019. We measured the effect of a simulated intrauterine sound on changes in the behavioral and physiological parameters of pain (heart rate, SPO_2_) caused by heel lance that was measured 5 min before the intervention, during the sampling, and 5 min after the procedure. We measured behavioral pain by video recording the infants’ faces and then the scoring neonatal infant pain scale (NIPS). Heart rate and SPO_2_ were measured using a pulse oximeter device. The data were analyzed using analysis of variance (ANOVA) and independent *t*‑test in SPSS software version 20.0.

**Results:**

Eighty infants were randomized (40 in each group). Mean scores NIPS during and after intervention were in the intervention group (3.55 ± 0.84, 95% CI: 3.30–3.80(, and (1.15 ± 0.84, 95%: 0.95–1.35) and in the control group (5.57 ± 0.95, 95% CI:5.30–5.85) and (3.00 ± 0.98) respectively. There were significant differences in scores of NIPS between the two study groups during (*p* < 0.001) and five min after heel lancing (*p* < 0.001). Mean scores of heart rate in the three phases of before, during, and five min after the intervention were respectively in the intervention group (127.57 ± 4.45, 95% CI:126.27-128.99), (131.07 ± 6.54, 95% CI:129.20-133.22), (128.45 ± 5.15, 95% CI:127.02-130.07) and in the control group (128.67 ± 4.57, 95% CI:127.32-130.07), (136.07 ± 7.24, 95% CI:133.90-138.37), and (132.42 ± 6.47, 95% CI:130.37-134.49). There were significant differences in heart rate between the intervention and the control group during (*p* = 0.002) and five min after the heel lance (*p* = 0.003). Mean scores of SPO_2_ in the three phases of baseline, during, and five min after the intervention were respectively in the intervention group (96.72 ± 0.93, 95% CI:96.42-97.00), (91.47 ± 1.46, 95% CI:91.05–91.92), (94.17 ± 1.03, 95% CI:93.22-94.00) and in the control group (96.6 ± 0.84, 95% CI:96.35–96.85), (91.5 ± 1.24, 95% CI:91.12–91.87), and (93.60 ± 1.27, 95% CI:93.85–94.50).

**Conclusion:**

This study showed that the simulated intrauterine sound reduces the behavioral pain and heart rate in the intervention group during and after heel lance. These results suggest using the method during the painful heel lancing to reduce pain parameters in preterm infants.

## Introduction

The incidence of preterm birth is a worldwide health concern in developing and developed countries. In addition, preterm birth has not significantly declined over the years. Preterm infants are vulnerable and at high risk for altered developmental disabilities and impaired neurodevelopmental outcomes [1]. Pain is experienced by all preterm infants in the neonatal intensive care unit (NICU) as they are exposed to many diagnostic and therapeutic procedures that are necessary for their survival.

Exposure of infants to acute procedural pain is inevitable and most of the time is created as a result of the medical procedures within the first day(s) after birth. According to previous studies, hospitalized preterm neonates may undergo up to 17 painful procedures per day [2–4], most of which are heel lancing. Poorly treated pain during the neonatal period may lead to negative long-term consequences. Recent studies have reported that painful procedures experienced by premature infants may lead to neurological and behavioral changes [5]. Also, physiologic responses to painful stimuli in preterm infants are manifested as acute increases in the heart rate, blood pressure, and intracranial pressure, as well as decreased arterial oxygen saturation. In addition, pain-related stress is associated with alterations in both early and later developmental outcomes [6]. Increased numbers of repeated painful interventions such as tissue-breaking procedures during NICU have been associated with poorer cognitive, motor, and behavioral neurodevelopmental outcomes in infancy [7]. Therefore, neonatal pain control is essential in newborn care [8]. Many epidemiological studies have reported the most commonly performed invasive painful procedures in premature infants i.e., heel lance [9–12].

Heel lancing is a routine procedure for capillary blood sampling for different diagnostic and therapeutic purposes for example screening tests to identify metabolic defects or measuring blood glucose in neonates. Heel‑prick sampling is a painful and stressful procedure in neonatal wards for diagnostic tests. Reaction to pain due to heel prick significantly differed from venous blood sampling and infants experienced more intense pain [13].

Many studies evaluated the effect of different nonpharmacological methods in preterm infants’ pain control, for example, mother-driven interventions such as auditory stimulations, maternal sounds or heart sound and skin-to-skin care [14–17], and other methods such as music [18, 19], types of noises [20], nutritive and non-nutritive sucking [21, 22], oral glucose solutions and small volumes of sweet solutions with or without non-nutritive sucking [23–26] to alleviate different types of procedural pain. Also, several studies were performed to assess the effect of other nonpharmacological methods for controlling pain caused by heel lancing. Breastfeeding, if possible, feasible and culturally acceptable, skin-skin care and other comfort measures are known to reduce pain [8, 24, 25, 27, 28]. However, Cruz et al. in their systematic review study reported that painful procedures were performed frequently and often with inadequate pain management. Unlike neonate clinical factors, organizational factors may be modified to promote a context of care more favorable to pain management [4]. Unfortunately, despite the existence of the mentioned strategies to reduce pain in preterm infants, in neonatal departments of hospitals in Iran, these techniques are not used to relieve the procedural pain caused by heel piercing. Due to various reasons such as the lack of adequate nurse staff and their high workload while providing special care, the restriction of entering mothers into the NICU departments, the withholding of oral feeding according to the physicians’ order, respiratory distress syndrome (RDS), and no permission to the consumption of oral glucose in very low birth weight infants. Therefore, it seems that methods that do not require the presence of a nurse or mother can minimize these limitations. One of these methods is the use of sensory stimulation. Jin et al. in a meta-analysis study reported that maternal voice reduces procedural pain in newborns. It is a beneficial approach to improve the physiological indicators of pain [29].

Auditory stimulus is a non-invasive method of sensory stimulation. It is a harmless procedure of pain relief by provoking feelings of security. A softy rhythmic music acts by distracting the attention of the person away from pain inducers. Also, it reduces adrenocorticotropic hormone (ACTH) levels and allows for the release of natural endorphins in the brain [30]. In addition, music stimulation in preterm infants creates higher oxygen saturation levels and the heart rate in newborns. Music can regulate respiratory rate in the normal range, and decrease stress levels, energy expenditure, and episodes of apneas and bradycardias [31]. The study of Patel et al. revealed that music therapy can have a positive effect on the various responses of preterm infants during painful procedures [32]. It seems that choosing a familiar and soothing voice that reminds the physiological attachment of the infant to the mother during the prenatal period can be a more suitable option to reduce the pain of preterm infants even in the absence of the mother during daily painful procedures. It can be a simulated voice of intrauterine sounds. To the best of our knowledge, there is only one study that assessed the effectiveness of a simulated intrauterine sound, which was the sleeping baby relaxing music, converted technologically to familiar intrauterine sounds on pain of venipuncture procedure in preterm infants [33]. No study was found to examine the efficacy of intrauterine sound during heel lancing in preterm newborns. Therefore, it is unknown whether the simulated intrauterine sound is effective in reducing acute pain associated with heel lancing in preterm newborns.

### Objectives

The present study was conducted to assess the effectiveness of a simulated intrauterine sound on behavioral and physiological indices of pain during heel lancing for capillary blood sampling to perform screening program tests.

## Methods

### Study design

This was a double‑blind, randomized clinical trial (RCT). The data were collected from September 23 to December 22, 2019. We assessed the effectiveness of playing a sound simulated with the intrauterine sounds in the prenatal period on behavioral and physiological indices of pain in preterm neonates. The independent variables for this study were the behavioral, and the physiological parameters of procedural pain that were measured during and immediately after a heel lance.

### Setting and participants

This study was conducted from October to December 2019 in the preterm neonatal department of Alzahra Hospital of Guilan University of Medical Sciences (GUMS), Rasht, Iran. The study population consisted of all preterm infants with 28–36 weeks gestational age who had a heel lance.

#### Inclusion criteria

Inclusion criteria were prematurity with gestational age 28-36weeks, birth age ≤ 1 week, hospitalization cared in a closed incubator, no intake anti convulsion and respiratory depressant drugs and analgesics, stable physiological status, normal audiometry examinations, birth weight < 2500 gr, the APGAR scores $\ge$7 at 1 and 5 min after birth.

#### Exclusion criteria

**Fig. 1:**
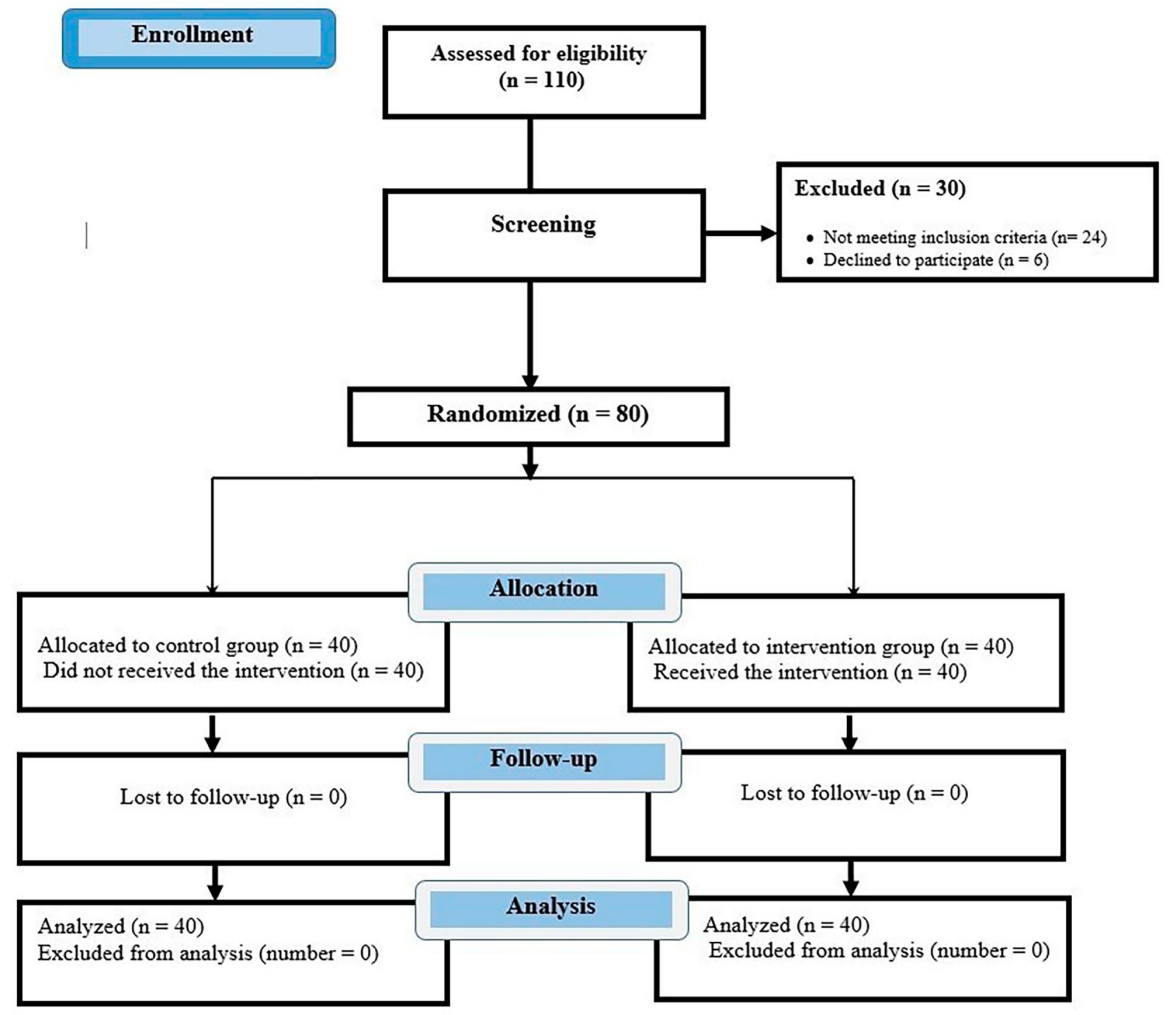
CONSORT flow diagram

 (1) Preterm infants with congenital anomalies; (2) those with intracranial hemorrhage and life-threatening situations; (3) unsuccessful puncture for blood collection at the first attempt. Figure 1. shows the dropout criteria and the follow-up of the study (Fig. 1).

### Sample size calculation

According to the sample size calculation formula involving the comparison of the two sets of means, the sample size was calculated based on the study of Alemdar et al. [34], such that the two-sided α = 0.05, 90% test power, the standard deviation of the primary outcome (behavioral pain score) in the intervention group (σ_1_ = 0.75) and the control group (σ_2_ = 0.55) after the intervention, d = 0.5 and with an attrition rate of 10. A 25% pain reduction was based on clinical importance and similar reductions used in sample size calculations of prior studies of pain reduction with needle stick research [35, 36].

$$n = \frac{{_{\left( {{z_{1 - \frac{\alpha }{2}}} + {z_{1 - \beta }}} \right)2(\sigma _1^2 + \sigma _2^2)}}}{{{d^2}}}$$

### Outcome measures

The primary outcome was behavioral pain response, and the secondary outcome was physiological pain responses in newborns during a heel stick. Physiological pain responses were HR and SpO2 levels evaluated during baseline (Before intervention) and heel lance (capillary blood sample) using a pulse oximetry device.

### Data collection

#### Instruments and measurements

##### Preterm Infant Demographic Information Form

This form included demographic data regarding the newborn’s gender, gestational age, current age, birth weight, current weight, head circumference, Apgar score (1 and 5 min after birth), type of feeding, time of the last feeding, type of delivery, number of multiple pregnancies, frequency of the heel lance procedure during intervention time.

##### Preterm Infant follow-up form

This form was designed to record the physiological measurements of infants (heart rate and oxygen saturation). Pain in neonates causes physiological changes such as increased heart rate and blood pressure and decreased oxygen saturation (SPO_2_) [5].

##### Neonatal infant Pain Scale (NIPS)

The Neonatal Infant Pain Scale (NIPS) was used to detect behaviors that are described as the infants’ pain or distress. In 1993, NIPS was developed by Lawrence et al. to assess the behavioral pain responses of neonatal infants [37]. It is comprised of 6 behavioral pain indicators containing changes in facial expression, arm movement, leg movement, crying, state of consciousness, and breathing pattern. Each indicator is scored with 0 or 1 (except “cry,” which has three possible descriptors and is scored with a 0, 1 or 2). A score greater than 3 indicates pain. To fully assess each indicator, the infants were observed for one minute in two phases before and after the intervention. NIPS is a tool with a final total score range of 0 to 7, and higher scores indicate an increase in the severity of pain. In this study, we calculated the reliability of NIPS on the 30 preterm infants in a pilot study. The inter-rater reliability was obtained above *r* = 0.87 before and during the study by scoring NIPS by the two nurse raters.

### Sampling

In this study, the eligible infants were randomly assigned through block randomization into two the experimental group (infants who were exposed to the simulated intrauterine sound) and the control group (infants who received routine care). The infants were entered into the study by convenience sampling method. The sampling process was carried out according to the principles of randomization in clinical trial studies in the CONSORT Checklist which had the three steps of creating a random sequence, concealing the random allocation, and implementing the randomization process carefully. Using the random block allocation method with blocks of four the samples were placed in the intervention and control groups. In this regard, at first, the letter A was assigned to the intervention group and letter B was assigned to the control group, and then were written six different possible placements of these two letters in blocks 4 including ABBA, ABAB, BAAB, BABA, BBAA, AABB in the separate sheets. For the concealment process, these 6 sheets were placed in sealed envelopes, and to determine random sequence, one of the envelopes was selected each time (10 selections for each study group with again replacement) and based on the sequence of the block inside the envelope, the sampling was done.

### Intervention

Before the data collection, the main researcher interviewed the parents of eligible infants to inform them about the study’s aim, and written consent was obtained from them. Then, a hearing screening test based on auditory brain stem responses was scheduled, and the participants entered the study according to the inclusion criteria. Before the intervention, the demographic questionnaires were completed, and the newborns were randomly assigned to the intervention or control group.

All heel lance procedures were performed by the same experienced nurse who worked on the day shift (who had a 10-year NICU experience). To collect the capillary blood sample, the preterm infants remained in the supine position. The nurse lightly cleaned the skin of the infants’ heel on their left foot using an alcoholic cotton pad, and blood sampling was done by inserting a disposable lancet 24-gauge needle. After removing the lancet needle, a dry cotton pad was placed on the insertion site. The duration of the blood sampling was a maximum of 2 min for all the participants.

Data was collected by the main researcher who was a master’s student in pediatric nursing. A portable pulse oximeter device (NOVAMETRIX, USA) was used to continuously record the heart rate (HR) and oxygen saturation (SpO2) levels. The pulse oximeter probe was placed on the sole of the infant’s right foot, and the observed values were recorded from the screen of the pulse oximeter device. An assistant who was an experienced nurse and blinded to the study aim, recorded HR, SpO2 level, and behavioral signs of the pain using a video camera in the three stages: the basic stage included 5 min before the intervention, during the intervention (2 min), and 5 min after the intervention. However, this nurse was not blinded about interventions in the two groups. At the end of each day, another experienced nurse who was not attending at the time of sampling in the hospital and was blind to the study groups scored the physiological and behavioral changes of the infants by watching the recorded videos. Finally, after the calculation, she entered the mean HR and SpO2 into the preterm infant follow-up forms.

### Intrauterine sounds’ group

In this study, the intervention was the simulated intrauterine sound that the infants of the intervention group were exposed to. The sound was obtained from specific relaxing music similar to real heartbeat sounds converted technologically to familiar intrauterine sounds for the infants. The simulated intrauterine sound was played to the preterm infants in the incubator through a small speaker system (501 TG, China) and an MP3 player. Before the intervention, the sound volume was set by a digital sound-level meter (SL-4013, Australia) to 45 decibels. Each preterm infant in this group was exposed to the simulated intrauterine sounds from 5 min before the heel lance to 5 min after finishing blood sampling.

### Control group

The infants in the control group received the gentle touch, which is a technique to reduce stimulation and pain in newborns, during the intervention by the same experienced nurse who implemented all of the heel lances. Among the non-pharmacological methods, gentle touch is effective in relieving pain. It consists of applying light and gentle pressure on the body of the newborn to stimulate low-threshold afferent fibers that influence the brain, autonomic nervous system, blood flow, and respiratory rate. The technique also provides immediate positive effects (e.g., comfort, decreased level of motor activity, and deep sleep), attenuates brain activity during painful procedures, increases oxygen saturation, and decreases heart rate and crying time [38].

The researcher used a video camera to record the behavioral changes of infants for 5 min before, during, and 5 min after the procedure. Heart rates and SO2 were recorded from the pulse oximetry.

### Data analysis

Descriptive statistics (means, standard deviations) were used. Shapiro-Wilk test was used to assess the normality of distributions. Differences in the mean scores on the behavioral pain levels between the two groups were assessed using an independent t-test. χ2 test was used to analyze categorical variables. Also, two sample Kolmogorov-Smirnov (KS) test was used for comparison of the homogeneity of the quantitative variables in the two study groups. Repeated measure ANOVA was used to assess the trend of within-group changes in the NIPS, heart rate and SPO2 in the three study phases. A *p*-value < 0.05 was considered statistically significant. The SPSS software version 20.0 was used for the data analysis.

## Results

Of 110 infants who were included in this study, 24 infants did not meet inclusion criteria and 6 parents declined to participate in the study before randomization. Finally, 80 infants participated in the study. The mean scores of gestational ages of the newborns in the intervention and the control groups were 34.62 ± 1.39 and 34.57 ± 1.21 respectively. There was no significant difference between the demographic characteristics of the two study groups before the intervention (*p* > 0.05), and the two groups were homogenous about all the considered variables (Table 1).

**Table 1:** Comparison of demographic characteristics of the preterm infants in the intervention and the control groups

Variables		*P*	Control group	Intervention group	
			Mean ± SD/ n (%)		
Gestational ages (weeks)		0.75 *	34.57 ± 1.21	34.62 ± 1.39	
Birth weight (gr)		0.91*	1972.25 ± 431.70	1999.25 ± 434.10	
After weight		0.91*	1957.87 ± 425.22	1991.12 ± 426.68	
Birth head circumference (Cm)		0.75*	34.35 ± 1.05	34.50 ± 1.10	
1-min APGAR score		1.00 *	8.22 ± 0.97	8.22 ± 1.02	
5- min APGAR score		1.00 *	9.12 ± 0.82	9.10 ± 0.84	
Duration of hospitalization		1.00 *	3.07 ± 1.30	2.97 ± 1.14	
Times of heel lance		1.00 *	2 ± 0.87	2.05 ± 0.90	
Time duration of the last feeding(min)		0.98 *	38.37 ± 4.58	39 ± 4.26	
Gender	Girl	0.82 **	17(42.5)	18(45)	
	Boy		23 (57.5)	22(55)	
Twins	Yes	0.64 **	3(7.5)	2(5)	
	No		37(92.5)	38(95)	
Type of feeding	Breast feeding	0.43 **	61(72.5)	32(80)	
	Bottle feeding		19(27.5)	8(20)	
Type of delivery	Vaginal delivery	0.39 **	34(85)	31(77.5)	
	Cesarean		6(15)	9(22.5)	

* Two sample Kolmogorov-Smirnov (KS)

** Chi square

Mean scores NIPS during and after intervention were in the intervention group (3.55 ± 0.84, 95% CI: 3.30–3.80(, and (1.15 ± 0.84, 95%: 0.95–1.35) and in the control group (5.57 ± 0.95, 95% CI:5.30–5.85) and (3.00 ± 0.98) respectively. There were significant differences in scores of behavioral pain scores between the two study groups before, during and five min after heel lancing (*p* < 0.001).

Mean scores of heart rate in the three phases of before, during, and five min after the intervention were respectively in the intervention group (127.57 ± 4.45, 95% CI:126.27-128.99), (131.07 ± 6.54, 95% CI:129.20-133.22), (128.45 ± 5.15, 95% CI:127.02-130.07) and in the control group (128.67 ± 4.57, 95% CI:127.32-130.07), (136.07 ± 7.24, 95% CI:133.90-138.37), and (132.42 ± 6.47, 95% CI:130.37-134.49). Mean scores and standard deviation of SPO_2_ in the three phases of baseline, during, and five min after the intervention were respectively in the intervention group (96.72 ± 0.93, 95% CI:96.42-97.00), (91.47 ± 1.46, 95% CI:91.05–91.92), (94.17 ± 1.03, 95% CI:93.22-94.00) and in the control group (96.6 ± 0.84, 95% CI:96.35–96.85), (91.5 ± 1.24, 95% CI:91.12–91.87), and (93.60 ± 1.27, 95% CI:93.85–94.50) (Table 1).

The mean ± SD of NIPS in the intervention and the control groups was 0.00 ± 0.00 at baseline. There were significant differences in scores of the NIPS between the intervention and the control group during heel lance (*p* < 0.001) and 5 min after the procedure(*p* < 0.001). On the other hand, the repeated measures ANOVA showed no significant difference between the intervention and control groups before, during and after the intervention regarding the mean SPO_2_ of the infants. However, increasing the SPO_2_ was significantly lower in the intervention group than in the control group 5 min after the heel lance (*p* = 0.03). There were significant differences in the heart rate between the intervention and the control group during (*p* = 0.002) and 5 min after the heel lance (*p* = 0.003) (Table 2).

**Table 2:** Comparison of mean scores of NIPS, SPO2, and heart rate before, during and after heel lance in the intervention and the control groups

Variables	Time	Intervention group	(95%) Confidence Interval		Control group	(95%) Confidence Interval		F	t	df	P*
		Mean ± SD	Lower	Upper	Mean ± SD	Lower	Upper				
NIPS	Before heel lance	0.00 ± 0.00	0.00	0.00	0.00 ± 0.00	0.00	0.00	-	-	78	0.001
	During heel lance	3.55 ± 0.84	3.30	3.80	5.57 ± 0.95	5.30	5.85	0382	-10.230	78	0.001
	After heel lance	1.15 ± 0.84	0.95	1.35	3.00 ± 0.98	2.70	3.30	5.004	-9.844	78	0.001
SPO_2_ (%)	Before heel lance	96.72 ± 0.93	96.42	97.00	96.60 ± 0.84	96.35	96.85	0.566	5.629	78	0.53
	During heel lance	91.47 ± 1.46	91.05	91.92	91.50 ± 1.24	91.12	91.87	0.916	-0.082	78	0.93
	After heel lance	93.60 ± 1.27	93.22	94.00	94.17 ± 1.03	93.85	94.50	2.441	-2.212	78	0.03
Heart rate (min)	Before heel lance	127.57 ± 4.45	126.27	128.99	128.67 ± 4.57	127.32	130.07	0.033	-1.089	78	0.27
	During heel lance	131.07 ± 6.54	129.20	133.22	136.07 ± 7.24	133.90	138.37	0.501	-3.240	78	0.002
	After heel lance	128.45 ± 5.15	127.02	130.07	132.42 ± 6.47	130.37	134.49	2.732	-3.036	78	0.003

* Independent t-test

The repeated measure ANOVA in assessing the trend of the changes in scores of NIPS at different times showed significant differences in the effect of time (*p* < 0.001), and group (*p* < 0.001) between the intervention and the control groups. The trend of changes in the SPO_2_ showed that the main effect of time (*p* < 0.001) was statistically significant in the two study groups. Also, there were significant differences in the trend of the heart rate scores in the effect of time (*p* < 0.001), and group (*p* < 0.006) between the intervention and the control groups (Table 3). The CONSORT diagram shows the steps of the study (Fig. 1).

**Table 3:** Comparison of trend of changes in mean scores of NIPS, SPO2 and heart rate in 5 min before, during, and 5 min after heel lance in the intervention and the control groups

Variables		Groups			
			Intervention	Control	P*
			Mean ± SD		
**NIPS**	Before heel lance		00.00 ± 00.00	00.00 ± 00.00	0.001
	During heel lance		3.55 ± 0.84	5.57 ± 0.95	0.001
	After heel lance		1.15 ± 0.66	3.00 ± 0.98	0.001
P**			0.001	0.001	
**SPO**_**2**_ **(%)**	Before heel lance		96.72 ± 0.93	96.60 ± 0.84	0.53
	During heel lance		91.47 ± 1.46	91.50 ± 1.24	0.93
	After heel lance		93.60 ± 1.27	94.17 ± 1.03	0.03
P**			0.001	0.001	
**Heart rate** **(min)**	Before heel lance		127.57 ± 4.45	127.67 ± 4.57	0.27
	During heel lance		131.07 ± 6.54	136.07 ± 7.24	0.002
	After heel lance		128.45 ± 5.15	132.42 ± 6.47	0.003
P**			0.001	0.001	

* Independent t-test

**Repeated measure ANOVA

## Discussion

The study aimed to investigate the effect of the simulated intrauterine sound on behavioral and physiological indices of pain during a heel lance on preterm infants. The study results showed the effectiveness of the simulated intrauterine sound on behavioral pain (NIPS scores) and the heart rate during and after heel lance in the experimental group. However, no significant difference was found in the SPO_2_ of the two study groups. Kurdahi Badr et al., in their study, showed that music provided via headphones to premature infants before, during and after a painful procedure had a significant effect on the infants’ pain scores but did not cause significant differences in their physiological measures [39]. Sarhangi et al. examined the impact of the mother’s heartbeat sound on physiological parameters and pain intensity after blood sampling in premature neonates. They indicated that a mother’s heartbeat sound as a nonpharmacological and safe intervention could reduce procedural pain in neonates [40]. As the mentioned studies showed the effectiveness of mild auditory stimulation in reducing pain, these results are consistent with the current research. Despite the strong evidence about the effectiveness of various non-pharmacological methods of reducing pain in infants, in the latest systematic review and meta-analysis studies in 2023, Garcia-Valdivieso et al. reported that non-pharmacological techniques such as breastfeeding, the mother-kangaroo method, oral sucrose or glucose and non-nutritive sucking are not effective in reducing neonatal pain-related parameters such as HR, PIPP scale and O2 saturation [41]. However, it seems the analgesic effectiveness of the strategies based on behavioral outcomes, is considered a more specific measure of pain than physiological parameters. This contradiction reveals the need for additional future research in the field of pain reduction methods in procedures with different pain intensities in newborns. We believe that the simulated intrauterine sound is an easy method that can be used in NICU wards, where the mothers are not attending. According to the evidence it is need to repeat the next studies about the more effective, easy and, applied strategies in neonatal pain management.

Parallel to the current study, the results of the study of Doheny et al. indicated that short-term improvements in the physiological stability of NICU infants included the heart rate using exposure to maternal biological sounds [42]. Other studies also showed the effect of mothers’ voices on the physiological reactions of premature infants [43, 44]. The results of Sajadian et al. revealed a decrease in both variables heart rate and respiratory rate during the voice period compared to the pre-voice period [45]. Valeri et al. in a systematic review of neonatal pain and the developmental outcomes in children born preterm showed greater numbers of painful procedures were associated with delayed postnatal growth, poor early neurodevelopment, high cortical activation, and altered brain development. In toddlers born with gestational age ≤ 32 wk, procedural pain reactivity-recovery scores were associated with negative affectivity temperament. Furthermore, poor quality of cognitive and motor development at 1 year of age and changes in cortical rhythmicity and cortical thickness in children at 7 years of age were associated with greater numbers of neonatal painful experiences [6]. However, the other systematic review study revealed inadequate pain management in painful procedures performed in neonates. The results stated the need for modification of organizational factors to promote a context of care more favorable to pain management [11].

The current study found no significant difference in the SPO_2_ levels between the intervention and control groups’ infants before, during, or after heel lance. Consistent with the current study results, Garunkstiene et al. reported no changes in the oxygen saturation levels of preterm neonates when subjected to their live or recorded maternal voice [3]. However, the study of Alemdar et al. to identify the effect of covering the eyes and playing the intrauterine sounds on premature infants’ pain and physiological parameters during venipuncture revealed that no significant difference was found in NIPS score between the intervention and control groups during venipuncture. In addition, no significant difference was found between the intervention and control groups in physiological parameters before, during, and after the intervention [33]. The results are inconsistent with the results of the current study on the base of the heart rate changes and consistent on the base of changes in the mean score of SPO_2_. It seems these different results are due to the intervention time interval and the variety of gestational ages of the participating infants. In addition, we used NIPS, which is a valid and reliable tool only for detecting behavioral pain scores. Also, we measured the physiological parameters with a pulse oximetry device which is an objective method.

Courtois et al. in their study showed that heel stick was very frequently performed in NICUs. Although most heel sticks were performed with analgesia, this was not systematic. The high frequency of this procedure and the known adverse effects of repetitive pain in neonates should encourage the search for safe, fast, and effective strategies to reduce their number and the adverse side effects [10].

Another study was performed to identify, characterize, and summarize research evidence on parent-delivered pain-relieving interventions in neonatal care where the parents themselves deliver the pain management. This review study highlighted the efficacy and advantages of involving parents in neonatal pain management such as skin-to-skin contact and breastfeeding. In these parent-delivered interventions which are consistent with modern family-integrated care, the parents are the mediator of pain relief. This study also confirmed the synergistic effects of combining these interventions [46].

In this regard, Canadian pain standards also recommended strategies that make better pain management in neonates through multimodal pain management methods. Multimodal pain management uses physical, psychosocial, nonpharmacological and pharmacological strategies to prevent and manage acute and chronic pain. Breastfeeding, oral sucrose, and kangaroo care or infants comfort positioning, facilitated tucking, or touch techniques to reduce stimulation, such as minimizing harsh lighting and noise, joint and tissue protection and positioning strategies such as braces, splints, or orthotics, and seating assessment, assistive or adaptive devices and mobility aids and thermal applications are examples the recommended methods [47].

Some limitations to this study should be noted. First, this study was conducted in a single center in a hospital setting. Although we controlled the environmental noises as much as possible, the hospital sounds can be a limitation in this study. The present study applied an easy and feasible method to relieve pain caused by the heel lance for capillary blood sampling in premature infants. It is suggested that further studies use this method as well as its combination with the mentioned known methods of non-pharmacological pain relief in venous or arterial blood sampling in term and preterm infants. Also, considering the inadequacies in the implementation of pain-reducing methods in hospitalized infants in Iran, it is suggested that next studies be conducted in the field of how to implement and use pain-reducing strategies in hospitalized infants.

Appropriate assessment and measurement of pain can help to the clinical judgement of pediatric nurses and Prevention of adverse effects of pain in infants [48].

## Conclusions

This study was conducted to identify the effects of playing simulated intrauterine sounds on pain and the physiological parameters during heel lance in premature infants. We found a significant difference between the intervention and control groups in NIPS and heart rate scores during and after heel lance. In addition, no significant difference was found in the SPO_2_ parameter of the two study groups before, during, and after heel lance. These results suggest the effectiveness of playing intrauterine sounds during a painful procedure to reduce pain parameters in preterm infants.

## Data Availability

The data that support the findings of this study are available from the corresponding author upon reasonable request.
